# Fludarabine-treosulfan compared to thiotepa-busulfan-fludarabine or FLAMSA as conditioning regimen for patients with primary refractory or relapsed acute myeloid leukemia: a study from the Acute Leukemia Working Party of the European Society for Blood and Marrow Transplantation (EBMT)

**DOI:** 10.1186/s13045-019-0727-4

**Published:** 2019-04-25

**Authors:** Francesco Saraceni, Myriam Labopin, Arne Brecht, Nicolaus Kröger, Matthias Eder, Johanna Tischer, Hélène Labussière-Wallet, Hermann Einsele, Dietrich Beelen, Donald Bunjes, Dietger Niederwieser, Tilmann Bochtler, Bipin N. Savani, Mohamad Mohty, Arnon Nagler

**Affiliations:** 1 Department of Internal Medicine and Hematology, AV3, ASUR Marche, Macerata, Italy; 2 EBMT Paris study office- CEREST-TC, Paris, France, Department of Haematology, Saint Antoine Hospital, Paris, France; 3 Deutsche Klinik fuer Diagnostik, KMT Zentrum, Wiesbaden, Germany; 4 University Hospital Eppendorf, Bone Marrow Transplantation Centre, Hamburg, Germany; 5 Department of Haematology, Hemostasis, Oncology and Stem Cell Transplantation, Hannover Medical School, Hannover, Germany; 6 Klinikum Grosshadern Med. Klinik III, Munich, Germany; 7 Centre Hospitalier Lyon Sud, Pavillon Marcel Bérard -Bat 1G, Service Hematologie, Lyon, France; 8 Universitaetsklinikum Wuerzburg, Med. Klinik und Poliklinik II, Wuerzburg, Germany; 9 University Hospital, Dept. of Bone Marrow Transplantation, Essen, Germany; 10 Klinik fuer Innere Medzin III, Universitätsklinikum Ulm, Ulm, Germany; 11 Division of Haematology & Oncology, University Hospital Leipzig, Leipzig, Germany; 12 University of Heidelberg, Medizinische Klinik u. Poliklinik V, Heidelberg, Germany; 13 Vanderbilt University Medical Center, Nashville, TN USA; 14 Department of Haematology, Saint-Antoine Hospital, Paris, France; 15 Department of Bone Marrow Transplantation, Chaim Sheba Medical Center, Tel-Hashomer, Israel; 16 Acute Leukemia Working Party – European Society for Blood and Marrow Transplantation, Paris, France

**Keywords:** Acute myeloid leukemia (AML), Active disease, Allogeneic transplantation, Sibling donor (MSD), Unrelated donor (UD), Conditioning regimen, Fludarabine-treosulfan (FT), Thiotepa-busulfan-fludarabine (TBF), Fludarabine, intermediate dose Ara-C, amsacrine, total body irradiation/busulfan, cyclophosphamide (FLAMSA)

## Abstract

**Background:**

Limited data is available to guide the choice of the conditioning regimen for patients with acute myeloid leukemia (AML) undergoing transplant with persistent disease.

**Methods:**

We retrospectively compared outcome of fludarabine-treosulfan (FT), thiotepa-busulfan-fludarabine (TBF), and sequential fludarabine, intermediate dose Ara-C, amsacrine, total body irradiation/busulfan, cyclophosphamide (FLAMSA) conditioning in patients with refractory or relapsed AML.

**Results:**

Complete remission rates at day 100 were 92%, 80%, and 88% for FT, TBF, and FLAMSA, respectively (*p* = 0.13). Non-relapse mortality, incidence of relapse, acute (a) and chronic (c) graft-versus-host disease (GVHD) rates did not differ between the three groups. Overall survival at 2 years was 37% for FT, 24% for TBF, and 34% for FLAMSA (*p* = 0.10). Independent prognostic factors for survival were Karnofsky performance score and patient CMV serology (*p* = 0.01; *p* = 0.02), while survival was not affected by age at transplant. The use of anti-thymocyte globulin (ATG) was associated with reduced risk of grade III–IV aGVHD (*p* = 0.02) and cGVHD (*p* = 0.006), with no influence on relapse.

**Conclusions:**

In conclusion, FT, TBF, and FLAMSA regimens provided similar outcome in patients undergoing transplant with active AML. Survival was determined by patient characteristics as Karnofsky performance score and CMV serology, however was not affected by age at transplant. ATG appears able to reduce the incidence of acute and chronic GVHD without influencing relapse risk.

**Electronic supplementary material:**

The online version of this article (10.1186/s13045-019-0727-4) contains supplementary material, which is available to authorized users.

## Background

Allogeneic hematopoietic stem cell transplant is the only potentially curative option for patients with acute myeloid leukemia (AML) in primary induction failure or refractory relapse. This population represents a big challenge for transplant physicians; nevertheless, according to recent evidence [1, 2], long-term survival can be achieved in about one third of patients undergoing transplant with active leukemia, and recent recommendations support prompt transplant in this setting, avoiding further chemotherapy [3]. The choice of the conditioning regimen is of vast importance in these fragile patients, as the need for powerful cytoreduction should not negate an acceptable toxicity profile of the protocol [4]. Historically, regimens employed in this setting included mainly standard myeloablative protocols based on alkylators or total-body irradiation (TBI) [5–7]. More recently, alternative strategies have been developed. The sequential fludarabine, intermediate dose Ara-C, amsacrine, total body irradiation/busulfan, cyclophosphamide (FLAMSA) regimen, designed by Kolb and colleagues in the early 2000s [8, 9], has shown promising outcome and currently represents one of the most widely employed protocols in this setting. On the other hand, the relentless effort of transplant physicians to temper conditioning toxicity while retaining a significant myeloablative power recently prompted the design of novel regimens, taking advantage in time of rather old drugs like thiotepa or treosulfan or combining two alkylators at reduced doses. The combination of thiotepa, busulfan, and fludarabine (TBF) was initially proposed as a preparative regimen for cord blood transplant [10]; subsequently, it has demonstrated excellent anti-leukemic activity in haploidentical [11, 12], matched sibling donor (MSD) and unrelated donor (UD) transplant [13, 14]. An additional option is represented by the association of fludarabine and treosulfan (FT), which has been intensely investigated in the last decade [15–18]. Preliminary results of a prospective randomized trial demonstrated promising outcome following FT conditioning in patients with AML and MDS [19]. Furthermore, in a recent retrospective study comparing treosulfan with busulfan-based regimens in patients with active leukemia at the time of transplant, FT protocol resulted in improved outcome [20]. Given the lack of available reports analyzing and comparing the alternative conditioning protocols in patients with refractory or relapsed AML, we designed the current study to compare outcome of FT, TBF, and FLAMSA regimens in this particularly challenging setting.

## Methods

### Study design and data collection

This is a registry-based retrospective study. Data were provided and the study design was approved by the Acute Leukemia Working Party (ALWP) of the European Society for Blood and Marrow Transplantation (EBMT), in accordance with the EBMT guidelines for retrospective studies. EBMT is a voluntary working group of more than 600 transplant centers which are required to report all consecutive stem cell transplantations and follow-up once a year. Audits are routinely performed to determine the accuracy of the data. Since 1990, patients have been able to provide informed consent that authorizes the use of their transplant information for research purposes. The ALWP of the EBMT granted ethical approval for this study.

We included in the analysis AML patients older than 18 years, who had received FT, TBF, or FLAMSA as conditioning regimen for transplant from matched sibling donor (MSD) or unrelated donor (UD) as first transplant in active disease status (defined as > 5% bone marrow blasts or detectable blasts in peripheral blood at the time of transplant). Patients with primary refractory AML, first or second relapse were included in the analysis. Stem cell transplants were performed between January 2005 and December 2016, and all data were reported to the ALWP of the EBMT. All unrelated donors were HLA-matched (10/10) or mismatched at one HLA locus (9/10). Patients who received conditioning regimens including oral busulfan or T-depleted grafts were excluded.

### End-point definitions and statistical analysis

Non-relapse mortality (NRM) was defined as death from any cause in the absence of prior disease recurrence. Disease relapse was defined according to standard hematologic criteria. Leukemia-free survival (LFS) was defined as survival without relapse. Overall survival (OS) was calculated from the day of transplant until death from any cause or last follow-up. GVHD-free relapse-free survival (GRFS) was defined by the first of the following events: acute GVHD grades III to IV, extensive chronic GVHD, relapse, or death [21]. Patients with no event were censored at last contact. The cause of death was categorized according to standard criteria. The cause of death of patients who experienced relapse at any time before death was considered relapse related. Acute and chronic GVHD were graded according to standard criteria. All outcomes were measured from the time of stem cell infusion. Follow-up was estimated using the reverse Kaplan-Meier method. LFS, OS, and GRFS were estimated using the Kaplan-Meier method [22], whereas NRM, relapse, and GVHD were estimated using cumulative incidence analysis considering competing risks [23]. Univariate comparisons were performed using the log-rank test for LFS, OS, and GRFS and Gray’s test for GVHD, relapse incidence, and NRM. For all univariate analyses, continuous variables were categorized and the median used as a cut-off point. Multivariate analyses were performed using the Cox proportional hazards model. All factors differing significantly in distribution between the three groups or associated with one outcome were included in the Cox model. The FLAMSA group was used as the reference group in all comparisons. Results are expressed as hazard ratio (HR) with 95% confidence interval (CI). All *p* values were two-sided, and *p* <  0.05 were considered statistically significant. Statistical analyses were performed with SPSS 22.0 (IBM Corp., Armonk, NY) and R3.2.3 software packages(R Development Core Team, Vienna, Austria).

## Results

### Patient, disease, and transplant characteristics

Eight hundred and fifty-six patients fulfilled the inclusion criteria for the present analysis. Among them, 113 patients received FT, 112 TBF, and 631 received the FLAMSA regimen. Three hundred and sixty-two patients (42%) were transplanted from a MSD, 347 (41%) from a 10/10 UD, and 147 (17%) from a 9/10 UD. The FLAMSA protocol was busulfan- or TBI-based in 32% and 68% of the patients, respectively. Busulfan total dose was 6.4 mg/kg in 210 patients (157 FLAMSA, 53 TBF) and 9.6 mg/kg in 61 patients (8 FLAMSA, 53 TBF), while it was 12.8 mg/kg in 43 patients (37 FLAMSA, 6 TBF). In the group receiving TBI as part of the FLAMSA regimen, the dose was 4 Gy for all patients. Treosulfan dose was 30 mg/m^2^, 36 mg/m^2^, or 42 mg/m^2^ in 9, 21, and 83 patients, respectively. Anti-thymocyte globulin (ATG) administration was more frequent in FLAMSA as compared to TBF and FT cohorts (88%, 58%, and 39%, respectively, *p* < 10^−3^). The median year of transplant was 2011, 2015, and 2010 for FT, TBF, and FLAMSA, respectively (*p* < 10^−3^). The FT group included significantly older patients compared to the TBF and FLAMSA cohorts (median age 58, 52, and 52 years, respectively, *p* < 10^−3^). Cytogenetic data were available in 56% of the patients; among them, 6% of patients were with favorable, 63% with intermediate, and 31% with adverse cytogenetics, with no significant difference between the three groups. Cytomegalovirus (CMV) serology of donor and patient differed between the three cohorts (*p* < 10^−3^). Disease status (primary refractory or relapsed AML), Karnofsky performance score (KPS), type of donor, and donor/patient gender match did not differ between the groups. Donor lymphocyte infusions were administered to 101 (16%) patients in the FLAMSA group, 10 (9%) TBF recipients, and 14 (13%) patients within the FT cohort. Fifty-seven patients in the FLAMSA, 8 patients in the TBF, and 6 patients in the FT group received a second allogeneic transplant. Patient, disease, and transplant characteristics are summarized in Table 1. Detailed information on drug doses and post transplant cell therapy is provided in the supplementary material (Additional file 1).

**Table 1:** Patient, disease, and transplant characteristics

	FT	TBF	FLAMSA	*p*
Number (total, 856)	113	112	631	
Follow-up for survivors (months), median (95% CI)	53 (10–34)	16 (3–10)	53 (95% CI 4–35)	< 0.001
Age of patient at HSCT (years), median (range) (IQR)	58 (21–76) (47–64)	52.1 (24.4–70.1) (38.1–61)	51.5 (18.1–76) (41.9–59.9)	0.001
Age of patient at HSCT (categorical), *n* (%)				0.028
< 50 years	35 (31%)	46 (41%)	280 (44%)	
≥ 50 years	78 (69%)	66 (59%)	351 (56%)	
Gender of patient, *n* (%)				0.09
Male	66 (59%)	71 (63%)	336 (53%)	
Female	47 (41%)	41 (37%)	295 (47%)	
Karnofsky performance status at SCT, *n* (%)				0.7
KPS < 80	14 (13%)	12 (11%)	61 (10%)	
KPS ≥ 80	95 (87%)	95 (89%)	523 (90%)	
Missing	4	5	47	
Cytogenetics, *n* (%)				0.6
Favorable	7 (6%)	2 (2%)	19 (3%)	
Intermediate	37 (33%)	38 (34%)	228 (36%)	
Adverse	20 (18%)	19 (17%)	112 (18%)	
Missing	49 (43%)	53 (47%)	272 (43%)	
Disease status, *n* (%)				0.2
Primary induction failure	73 (64%)	59 (53%)	344 (55%)	
First relapse	30 (27%)	44 (39%)	241 (38%)	
Second relapse	10 (9%)	9 (8%)	46 (7%)	
Year of transplant, median (range)	2011 (2005–2016)	2015 (2007–2016)	2010 (2005–2016)	< 0.001
Donor, *n* (%)				0.06
MSD	56 (49%)	54 (48%)	252 (40%)	
UD 10/10	44 (39%)	35 (31%)	268 (42%)	
UD 9/10	13 (12%)	23 (21%)	111 (18%)	
Donor/recipient sex mismatch, *n* (%)				0.8
F to M	19 (18%)	21 (19%)	124 (20%)	
No F to M	87 (82%)	91 (81%)	490 (80%)	
Stem cell source, *n* (%)				< 0.001
BM	4 (4%)	19 (17%)	15 (2%)	
PBSCs	109 (96%)	93 (83%)	616 (98%)	
CMV donor/recipient, *n* (%)				< 0.001
Donor−/Recipient−	22 (21%)	13 (12%)	167 (27%)	
Donor+/Recipient−	9 (8%)	8 (7%)	76 (12%)	
Donor−/Recipient+	21 (19%)	25 (23%)	140 (23%)	
Donor+/Recipient+	57 (52%)	61 (58%)	229 (37%)	
ATG used, *n* (%)				< 0.001
No	69 (61%)	46 (42%)	73 (12%)	
Yes	44 (39%)	64 (58%)	554 (88%)	

Some percentages do not add up to 100% because of rounding

*ATG* anti-thymocyte globulin, *BM* bone marrow, *CMV* cytomegalovirus, *FLAMSA* fludarabine, intermediate dose Ara-C, amsacrine, total body irradiation, cyclophosphamide sequential regimen, *KPS* Karnofsky performance status, *FT* fludarabine-treosulfan, *GVHD* graft-versus-host disease, *LFS* leukemia-free survival, *MAC* myeloablative, *MSD* matched sibling donor, *NRM* non-relapse mortality, *OS* overall survival, *PBSCs* peripheral blood stem cells, *RI* relapse incidence, *TBF* thiotepa-busulfan-fludarabine, *TBI* total-body irradiation, *UD* unrelated donor

### Engraftment, disease response, and graft-vs-host disease

Engraftment rate was 98%, 91%, and 95% with median time to neutrophil engraftment of 16, 15, and 14 days in the FT, TBF, and FLAMSA cohorts, respectively (*p* = 0.1; *p* = 0.02). Median time to platelet engraftment was 15 days in the TBF group and 14 days in FT and FLAMSA groups. Graft failure was observed in four patients in the FLAMSA group and in one patient in the TBF and FT groups each. Secondary graft rejection was observed in six patients in the FLAMSA group while in none of the others. Cumulative incidence of complete remission for patients that reached day 100 was 92%, 80%, and 88% for the FT, TBF, and FLAMSA groups, respectively (*p* = 0.13). Global incidence of grade II–IV and III–IV acute GVHD (aGVHD) was 28% and 11%, respectively. The incidence of grade II–IV aGVHD was similar between the three groups, being 24%, 29%, and 28% in FT, TBF, and FLAMSA, respectively (*p* = 0.7). Similarly, the incidence of grade III–IV aGVHD did not differ between the three cohorts, 10% for FT, 12% for TBF, and 11% for FLAMSA, respectively (*p* = 0.9). Frequencies of chronic GVHD (cGVHD) and severe cGVHD in the global population were 27% and 12%, respectively. By univariate analysis, the cumulative incidence of cGVHD and severe cGVHD was similar in the three groups, being 33%, 26%, and 26% (*p* = 0.4) and 13%, 19%, and 11% (*p* = 0.5) for FT, TBF, and FLAMSA, respectively (Additional file 1). In multivariate analysis, the only factors associated with increased risk of developing aGVHD were transplant from mismatched unrelated donor and female/male donor/patient gender match. The use of ATG was independently associated with reduced risk of grade III–IV aGVHD and cGVHD (Table 2).

**Table 2:** Multivariate analysis of transplantation outcome

Outcome		HR	95% CI	*p*
RI	FLAMSA (ref)	1		
	TBF	0.9	0.6–1.4	0.6
	FT	0.8	0.5–1.2	0.2
	Age (per 10 years)	0.9	0.8–0.9	0.005
	Relapse vs prim. ref	1.3	1.1–1.6	0.01
	Patient CMV pos.	1.3	1.03–1.7	0.03
NRM	FLAMSA (ref)	1		
	TBF	1.5	0.8–2.7	0.17
	FT	1.2	0.7–2.1	0.5
	Age (per 10 years)	1.3	1.1–1.5	0.002
	MSD (reference)	1		
	UD 10/10	1.5	0.9–2.3	0.08
	UD 9/10	1.8	1.1–2.9	0.03
LFS	FLAMSA (ref)	1		
	TBF	1.1	0.7–1.5	0.7
	FT	0.9	0.6–1.3	0.6
	Patient CMV pos.	1.4	1.1–1.7	0.005
OS	FLAMSA (ref)	1		
	TBF	1.2	0.8–1.7	0.3
	FT	0.8	0.6–1.2	0.4
	KPS ≥ 80%	0.7	0.5–0.9	0.01
	Patient CMV pos.	1.3	1.1–1.6	0.02
GRFS	FLAMSA (ref)	1		
	TBF	0.9	0.7–1.4	0.9
	FT	0.8	0.6–1.07	0.13
	KPS ≥ 80%	0.7	0.5–0.9	0.01
	Patient CMV pos.	1.2	1.004–1.5	0.05
	ATG used	0.8	0.6–1.01	0.06
aGVHD III–IV	FLAMSA (ref)	1		
	TBF	0.9	0.4–2.1	0.8
	FT	0.7	0.3–1.6	0.4
	KPS ≥ 80%	0.5	0.3–1.02	0.06
	MSD (reference)	1		
	UD 10/10	1.6	0.8–2.9	0.16
	UD 9/10	3.6	1.9–6.9	< 0.001
	Female D to male R	1.7	1.01–2.9	0.045
	ATG used	0.4	0.2–0.9	0.018
cGVHD	FLAMSA (ref)	1		
	TBF	1.7	0.7–4.1	0.2
	FT	0.7	0.3–1.6	0.4
	Age (per 10 years)	0.8	0.7–0.9	0.03
	ATG used	0.4	0.2–0.8	0.006
Severe cGVHD	FLAMSA (ref)	1		
	TBF	1.4	0.6–3.3	0.4
	FT	0.6	0.2–1.3	0.2
	Donor CMV pos.	1.7	0.99–2.7	0.05
	ATG used	0.4	0.2–0.7	0.005

Hazard ratios of the three different conditioning regimens (FLAMSA as reference) and variables with *p* values below 0.05 are reported

*ATG* anti-thymocyte globulin, *BM* bone marrow, *CMV* cytomegalovirus, *FLAMSA* fludarabine, intermediate dose Ara-C, amsacrine, total body irradiation, cyclophosphamide sequential regimen, *KPS* Karnofsky performance status, *FT* fludarabine-treosulfan, *GVHD* graft-versus-host disease, *LFS* leukemia-free survival, *MSD* matched sibling donor, *NRM* non-relapse mortality, *OS* overall survival, *PBSCs* peripheral blood stem cells, *RI* relapse incidence, *TBF* thiotepa-busulfan-fludarabine, *UD* unrelated donor

### *NRM*, relapse, and survival

**Fig. 1:**
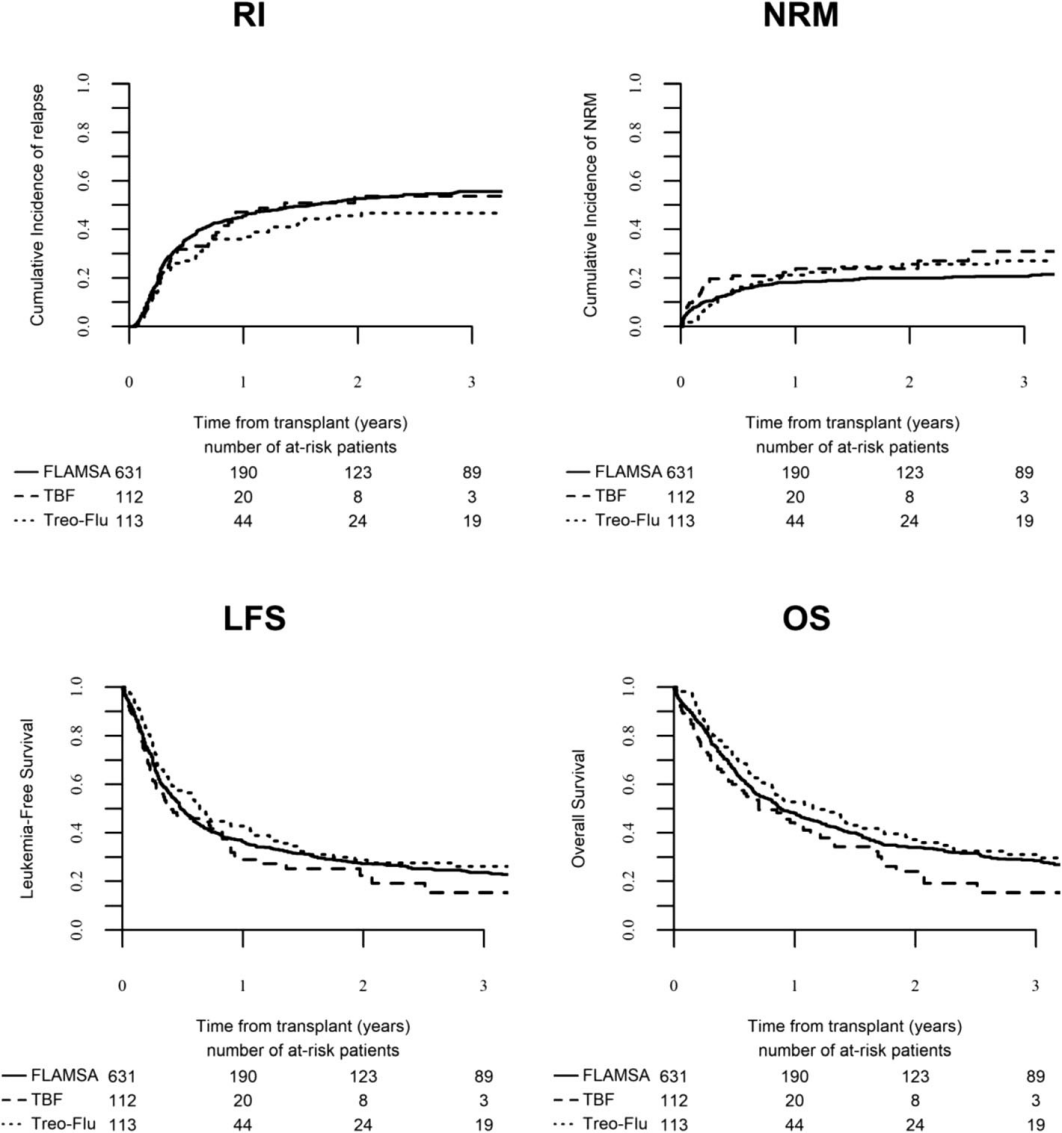
Transplant outcome following FT, TBF, and FLAMSA regimens. RI relapse incidence, NRM non-relapse mortality, LFS leukemia-free survival, OS overall survival. RI: *p*=0.33; NRM: *p*=0.24; LFS: *p*=0.28; OS: *p*=0.10

Global NRM rate was 7% at 100 days and 22% at 2 years. Six (5%) patients following FT, 14 (13%) following TBF and 40 (6%) following FLAMSA regimen died within 100 days. By univariate analysis, non-relapse mortality at 2 years was similar between the three groups: 26%, 24%, and 20% in FT, TBF, and FLAMSA, respectively (*p* = 0.24) (Fig. 1). In multivariate analysis, factors associated with increased NRM risk were older age and transplant from mismatched UD (Table 2). Leading causes of NRM were GVHD and infectious complications; the complete list of causes of death and their relative incidence are detailed in Table 3.

**Table 3:** Causes of death

	FT	TBF	FLAMSA
Total	75	67	410
Hemorrhage	2 (3%)	2 (3%)	4 (1%)
Failure/rejection	1 (2%)	0 (0%)	2 (1%)
Infection	11 (16%)	18 (27%)	73 (19%)
Interstitial pneumonitis	1 (1%)	2 (3%)	6 (2%)
GVHD	7 (10%)	7 (10%)	31 (8%)
Original disease	40 (59%)	27 (40%)	244 (63%)
VOD	0 (0%)	4 (6%)	8 (2%)
Other transplantation related	5 (7%)	7 (10%)	18 (5%)
Missing	7	0	21

*FLAMSA* fludarabine, intermediate dose Ara-C, amsacrine, total body irradiation, cyclophosphamide sequential regimen, *FT* fludarabine-treosulfan, *GVHD* graft-versus-host disease, *TBF* thiotepa-busulfan-fludarabine, *VOD* veno-occlusive disease

Cumulative incidence of relapse in the entire population was 52% at 2 years. By univariate analysis, 2-year relapse incidence was not statistically different between the three groups; 46%, 54%, and 53% for FT, TBF, and FLAMSA, respectively (*p* = 0.33). Multivariate analysis confirmed those results. Factors independently associated with higher risk of relapse were age at transplant, relapsed vs primary refractory AML, and patient CMV positive serology. Of note, the use of ATG did not influence relapse risk.

**Fig. 2:**
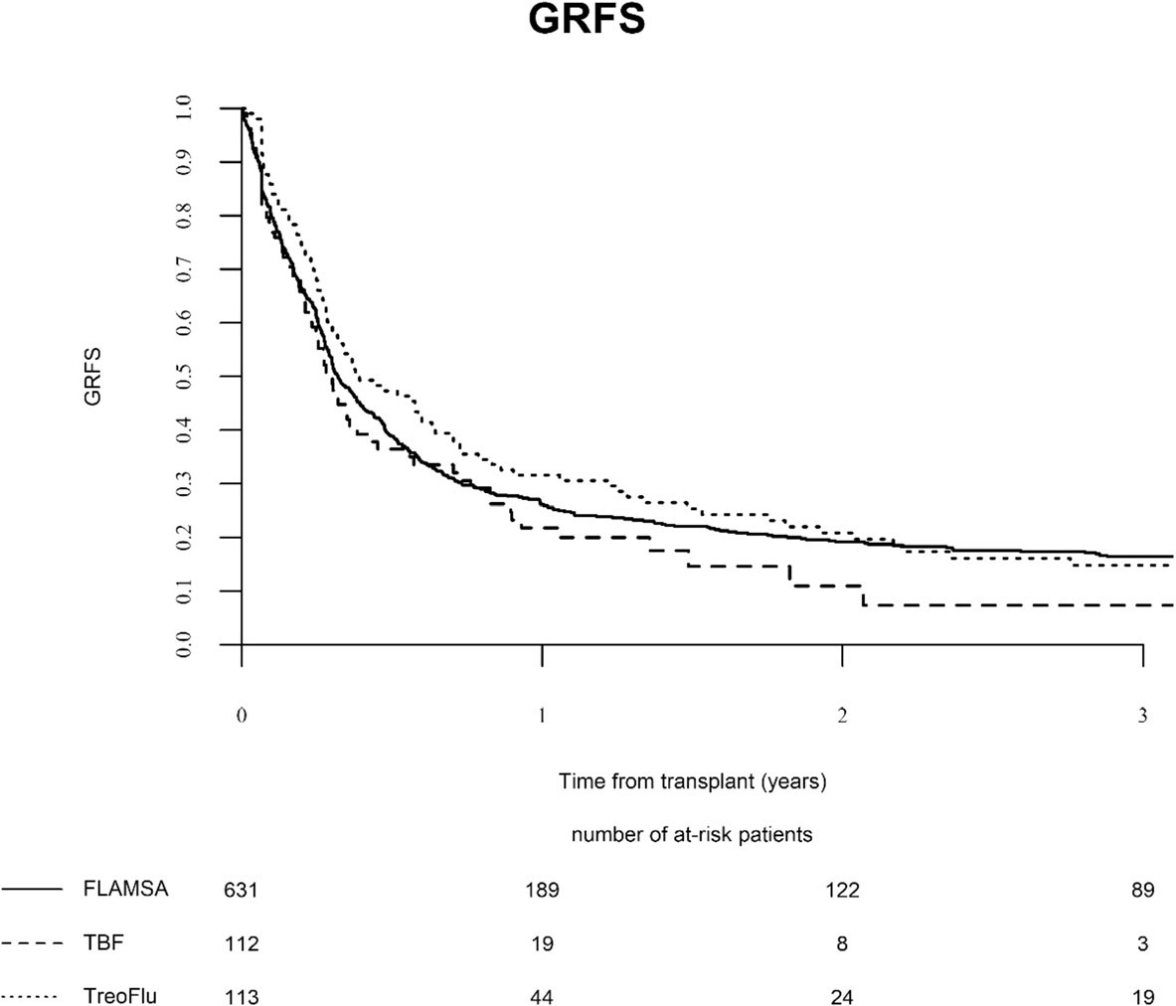
Graft-vs-host free, disease-free survival (GRFS) following FT, TBF, and FLAMSA regimens. *p*=0.15

Leukemia-free survival, overall survival, and GRFS in the global population were 27%, 34%, and 20%, respectively. Leukemia-free survival at 2 years was similar among the three groups: 29%, 22%, and 27% for FT, TBF, and FLAMSA, respectively (*p* = 0.28). Overall survival did not significantly differ as well, being 37% for FT, 24% for TBF, and 34% for FLAMSA (*p* = 0.10). In multivariate analysis, patient CMV positive serology was associated with inferior LFS. The factors predicting inferior OS were KPS lower than 80% and patient CMV positive serology. The composite endpoint GRFS at 2 years was 23%, 13%, and 20% for FT, TBF, and FLAMSA, respectively (*p* = 0.15) (Fig. 2). In multivariate analysis, KPS lower than 80% and patient CMV positive serology were independently associated with inferior GRFS.

## Discussion

Limited data is available to guide the choice of the conditioning regimen for patients with primary refractory or relapsed AML. We thus analyzed and compared the outcome of three commonly used conditioning regimens for active AML namely fludarabine-treosulfan, thiotepa-busulfan-fludarabine, and FLAMSA sequential regimen. Our results indicate global survival of 34% at 2 years; the type of conditioning protocol did not significantly affect survival, which was mostly determined by patient characteristics.

A major obstacle in transplanting patients with active leukemia is the high risk of non-relapse mortality; in fact, historical trials employing standard busulfan- or TBI-based regimens report a NRM rate of approximately 30–40% at day 100 after transplant [24–26]. In our study including patients up to 76 years of age, NRM at day 100 was around 5% following FT and FLAMSA and 13% after TBF, this difference being not statistically significant. Similarly, NRM at 2 years did not differ among the three regimens. It is important to highlight that the FT cohort included significantly older patients as compared to TBF and FLAMSA groups; in fact, 70% of FT patients were older than 50 years (median age of FT group, 58 years). Different strategies have been followed by researchers aiming to reduce mortality and improve the outcome of patients undergoing transplant with persistent leukemia. The design of the sequential FLAMSA regimen by the German group has represented a major breakthrough in this setting, combining promising anti-leukemic activity with acceptable NRM (about 22% at 2 years) [9]. On the other hand, some recent evidence suggests that redesigning standard myeloablative regimens could be an alternative strategy [1]. In fact, the Gruppo Italiano Trapianto di Midollo Osseo (GITMO) selected the TBF protocol as conditioning regimen for the GANDALF prospective trial, whose results have been recently presented, reporting a NRM rate of 35% at 2 years [27]. An alternative regimen is represented by the combination of fludarabine and treosulfan, which was shown to provide an interesting safety profile and promising outcome in patients undergoing transplant in remission [17] or with persistent leukemia [18, 20].

Importantly, the tolerability of the conditioning regimen should not compromise powerful antitumor activity in patients undergoing transplant with active AML. In the current study, we observed a complete remission rate at day 100 of about 90% following FT and FLAMSA, while CR rate was 80% after TBF, this difference being not statistically significant. Similarly, survival did not significantly differ among the three groups. Two-year survival rates were around 35% following FT and FLAMSA protocols; the latter was in accordance with the original report by the Munich group [8]. Conversely, TBF regimen was associated with a survival rate of 24% at 2 years, consistently with recent evidence from the GITMO trial employing the same protocol (OS at 2 years, 18%) [27]. Incidence of acute and chronic GVHD did not differ between the three regimens. In previous reports including patients with AML in remission, FT protocol has been associated with lower rates of GVHD as compared to busulfan-based regimens; we could not confirm this finding in our population of patients undergoing transplant with persistent leukemia [18, 28]. In fact, previous literature indicates a higher incidence of GVHD in patients transplanted with active disease in comparison to patients undergoing transplant in remission [29]. Nevertheless, we observed a tendency towards better GRFS following the FT protocol.

The rather large cohort included in the present study has allowed us to perform a multivariate analysis on factors predicting transplant outcome in the setting of active AML. Karnofsky performance score below 80% and patient positive CMV serology were strongly associated with poor survival. Further, patients with relapsed AML showed significantly higher risk of disease recurrence after transplant as compared to primary refractory AML. These findings are in accordance with a great body of previous evidence [30–32]. Transplant from mismatched UD predicted higher risk of grade III–IV aGVHD and non-relapse mortality, with no significant impact on survival. Interestingly, the use of ATG was associated with significantly lower incidence of both acute and chronic GVHD and a strong tendency towards better GRFS, with no influence on relapse rates. The benefit of ATG in terms of reduced incidence of GVHD is well established [33, 34]. However, as graft-versus-leukemia correlates with GVHD, there is a theoretic concern that the use of ATG could result in increased relapse rates, especially in patients undergoing transplant with active leukemia. Interestingly, in the historical ATG trial by GITMO, which included a high proportion of patients with active AML receiving transplant from unrelated donors, the use of ATG was associated with lower incidence of acute and chronic GVHD with no impact on relapse [35]. The findings of the present study are in line with these data. Furthermore, a recent report on patients with high risk AML undergoing transplant following a reduced intensity conditioning regimen including ATG confirmed no increased relapse risk [36]. Finally, it is of interest that in our cohort of patients aged up to 76 years, age at transplant did not influence survival, indicating that older age should not be taken as a criterion to withhold transplant in patients with active AML.

The limitations of the present study are mostly related to its retrospective design; in fact, limited information is available on the reason why a specific patient was allocated to a certain regimen. Further, scarce data on minimal residual disease status after transplant was available in the database; similarly, information on treatment administered after transplant other than cellular therapies (i.e., chemotherapy, target therapies, hypometilating drugs) was incomplete. Nevertheless, since a prospective randomized study comparing the three different conditioning protocols in patients with active AML has not been conducted yet and it is unlikely to be performed in the near future, we believe the results of the present analysis might serve to guide physicians practice in this very high-risk patients.

In conclusion, allogeneic transplant should be strongly considered in primary refractory and relapsed AML, as it is able to provide long-term survival in about one third of these patients. FT, TBF, and FLAMSA represent three possible alternative conditioning options in this setting, providing similar efficacy, toxicity, and survival. In fact, outcome was strongly affected by patient characteristics including Karnofsky performance score and CMV serology, while age should not be taken per se as a criterion to select patients for transplant. The use of ATG was associated with reduced incidence of severe acute and chronic GVHD without influencing relapse risk. Relapse remains the major cause of transplant failure; novel post-transplant strategies are thus in need to prevent disease recurrence in this extremely high-risk population.

## Additional file

Additional file 1: **Table S1.** Post-transplant cell therapy. **Table S2.** Drug doses in the three conditioning regimens (TBF, FT, FLAMSA). **Table S3.** Univariate analysis of ATG yes vs no stratified by conditioning regimen. (DOC 110 kb)

## Abbreviations

ALWP: Acute Leukemia Working Party
AML: Acute myeloid leukemia
BM: Bone marrow
EBMT: European Society for Blood and Marrow Transplantation
FLAMSA: Fludarabine, intermediate dose Ara-C, amsacrine, total body irradiation/busulfan, cyclophosphamide sequential regimen
FT: Fludarabine-treosulfan
GVHD: Graft-versus-host disease
LFS: Leukemia-free survival
MAC: Myeloablative
MSD: Matched sibling donor
NRM: Non-relapse mortality
OS: Overall survival
PBSCs: Peripheral blood stem cells
RI: Relapse incidence
TBF: Thiotepa-busulfan-fludarabine
TBI: Total-body irradiation
UD: Unrelated donor
