# Supplementary material for: Fludarabine-treosulfan compared to thiotepa-busulfan-fludarabine or FLAMSA as conditioning regimen for patients with primary refractory or relapsed acute myeloid leukemia: a study from the Acute Leukemia Working Party of the European Society for Blood and Marrow Transplantation (EBMT)
Source: J Hematol Oncol. 2019 Apr 25;12:44. doi: 10.1186/s13045-019-0727-4 (PMC6482556; doi:10.1186/s13045-019-0727-4)
Supplement: Supplementary file 1 — Table S1. Post-transplant cell therapy. Table S2. Drug doses in the three conditioning regimens (TBF, FT, FLAMSA). Table S3. Univariate analysis of ATG yes vs no stratified by conditioning regimen. (DOC 110 kb) [file 13045_2019_727_MOESM1_ESM.doc]

***Supplementary File***

**Table S1. Post transplant cell therapy**

- 1. Prophylactic/preemptive DLI:

|  | **TBF** | **FT** | **FLAMSA** |
| --- | --- | --- | --- |
| No DLI | 108 (93.4%) | 110 (97.9%) | 580 (91.9%) |
| Preemptive DLI | 4 (3.6%) | 3 (2.7%) | 51 (8.1%) |
| Total | 112 | 113 | 631 |

P=0.04

- 1. Treatment for relapse:

|  | **TBF** | **FT** | **FLAMSA** |
| --- | --- | --- | --- |
| no DLI/ no HSCT2 | 34 (75.6%) | 37 (68.5%) | 212 (69.5%) |
| DLI | 3 (6.7%) | 11 (20.4%) | 36 (11.8%) |
| HSCT2 | 5 (11.1%) | 6 (11.1%) | 43 (14.1%) |
| DLI + HSCT2 | 3 (6.7%) | 0 | 14 (4.6%) |
| **Total** | **45** | **54** | **305** |
| missing | 1 | 2 | 18 |

**Table S2. Drug doses in the three conditioning regimens (TBF, FT, FLAMSA)**

| **TBF** | Busulfan (mg/Kg) | median (range) | 9.6 (6.4-12.8) |
| --- | --- | --- | --- |
|  |  | 6.4 | 53 |
|  |  | 9.6 | 53 |
|  |  | 12.8 | 6 |
|  | Thiotepa (g) | median (range) | 10 (5-10) |
|  |  | 5 | 23 |
|  |  | 10 | 82 |
|  |  | missing | 7 |
|  | Fludarabine (mg/m2) | median (range) | 150 (90-180) |
|  |  | 90 | 21 |
|  |  | 120-130 | 6 |
|  |  | 150 | 75 |
|  |  | 160 | 4 |
|  |  | 180 | 6 |
| **FT** | Fludarabine (mg/m2) | median (range) | 150 (90-180) |
|  |  | 90 | 5 |
|  |  | 120-130 | 12 |
|  |  | 150 | 94 |
|  |  | 180 | 1 |
|  |  | missing | 1 |
|  | Treosulfan (g/m2) | median (range) | 42 (30-42) |
|  |  | 30 | 9 |
|  |  | 36 | 20 |
|  |  | 42 | 81 |
|  |  | missing | 3 |
| **FLAMSA** | Amsacrine (mg/m2) | median (range) | 400 (300-1000) |
|  |  | 300 | 15 |
|  |  | 400 | 427 |
|  |  | 500 | 5 |
|  |  | 600 | 27 |
|  |  | 700 | 66 |
|  |  | 800 | 56 |
|  |  | 1000 | 3 |
|  |  | missing | 32 |
|  | AraC (g/m2) | median (range) | 8 (2-8) |
|  |  | 2 | 9 |
|  |  | 4 | 24 |
|  |  | 6 | 20 |
|  |  | 8 | 486 |
|  |  | missing | 92 |
|  | Fludarabine (mg/m2) | median (range) | 120 (90-180) |
|  |  | 90 | 12 |
|  |  | 120 | 477 |
|  |  | 150 | 11 |
|  |  | 180 | 40 |
|  |  | missing | 91 |
|  | if TBI (n=377) | 4 Gy for all patients |  |
|  | if Bu (mg/Kg) | median (range) | 6.4 (5.4-12.8) |
|  | n=202 | 6.4 | 157 |
|  |  | 9.6 | 9 |
|  |  | 12.8 | 37 |
|  |  | missing | 51 |

Table S3. Univariate analysis of ATG yes vs no stratified by conditioning regimen

| **2y results** |  |  |  |  |  |
| --- | --- | --- | --- | --- | --- |
|  |  | **relapse** | **NRM** | **LFS** | **OS** |
| FLAMSA | no invivo TCD | 47.7%[35.2-59.3] | 12%[5.5-21.3] | 40.2%[28.2-52.2] | 41.7%[29.2-54.2] |
|  | invivo TCD | 53.7%[49.2-58] | 20.6%[17.2-24.2] | 25.7%[21.7-29.7] | 33.1%[28.7-37.4] |
|  | P value | 0.98 | 0.10 | 0.18 | 0.34 |
| TBF | no invivo TCD | 48.3%[30.7-63.7] | 25.2%[13.2-39.2] | 26.5%[11.6-41.4] | 20.6%[2.8-38.4] |
|  | invivo TCD | 58.4%[40.9-72.3] | 20.5%[11-32] | 21.2%[7.6-34.7] | 25.9%[12.1-39.7] |
|  | P value | 0.59 | 0.46 | 0.81 | 0.77 |
| Treo-Flu | no invivo TCD | 46.3%[33.8-57.9] | 26.1%[16-37.3] | 27.6%[16.6-38.6] | 36.7%[24.7-48.6] |
|  | invivo TCD | 45.5%[28.5-61] | 23.9%[12.2-37.7] | 30.7%[15.5-45.8] | 37.5%[21.1-54] |
|  | P value | 0.39 | 0.92 | 0.43 | 0.63 |

**Appendix (list of contributors)**

University Hospital, Essen, Germany; Helsinki University Central Hospital, Helsinki, Finland; Universitätsklinikum Dresden, Dresden, Germany; University of Freiburg, Freiburg, Germany; Hopital St. Louis, Paris, France; Deutsche Klinik für Diagnostik, Wiesbaden, Germany; University Hospital Eppendorf, Hamburg, Germany; Hannover Medical University, Hannover, Germany; University Hospital Leipzig, Leipzig, Germany; CHU Bordeaux, Pessac, France; Silesian Medical Academy, Katowice, Poland; Centre For Clinical Haematology, Birmingham, United Kingdom; Erasmus MC-Daniel den Hoed Cancer Centre, Rotterdam, Netherlands, The; Hopital E. Herriot, Lyon, France; Nottingham City Hospital, Nottingham, United Kingdom; University Medical Centre, Utrecht, Netherlands, The; Centre Pierre et Marie Curie, Alger, Algeria; University Hospital, Basel, Switzerland; Sezione di Ematologia, Perugia, Italy; University Hospital Gasthuisberg, Leuven, Belgium; Medizinische Klinik und Poliklinik, Ulm, Germany; Huddinge University Hospital, Huddinge, Sweden; Hopital Claude Huriez, Lille, France; Charles University Hospital, Pilsen, Czech Republic; Hopital de Purpan, Toulouse, France; Hopital Saint Antoine, Paris, France; University Medical Center St. Radboud, Nijmegen, Netherlands, The; Charité Universitätsmedizin Berlin, Berlin, Germany; Tel-Aviv University, Tel-Hashomer, Israel; King Faisal Specialist Hospital & Research Centre, Riyadh, Saudi Arabia; Leiden University Hospital, Leiden, Netherlands, The; Hôpital Henri Mondor, Creteil, France; CHU Nantes, Nantes, France; St. James Hospital Trinity College, Dublin, Ireland; University of Heidelberg, Heidelberg, Germany; Service d`Onco Hematologie, Strasbourg, France; Ospedali Riuniti di Bergamo, Bergamo, Italy; Klinikum Nürnberg, Nürnberg, Germany; Universität Tübingen, Tübingen, Germany; Hopital Jean Minjoz, Besancon, France; Hôpital Necker, Paris, France; Unité de transplantation et de thérapie cellulaire, Marseille, France; Royal Marsden Hospital, London, United Kingdom; GKT School of Medicine, London, United Kingdom; Klinikum Grosshadern, Munich, Germany; Policlinico San Matteo, Pavia, Italy; George Papanicolaou General Hospital, Thessaloniki, Thessaloniki, Greece; University of Münster, Münster, Germany; Erciyes Medical School, Kayseri, Turkey; Cliniques Universitaires St. Luc, Brussels, Belgium; Bologna University, S.Orsola-Malpighi Hospital, Bologna, Italy; Hopital A. Michallon, Grenoble, France; Gazi Universitesi Tip Fakültesi, Ankara, Turkey; University College London Hospital, London, United Kingdom; Univ.`La Sapienza`, Rome, Italy; CHU Lapeyronie, Montpellier, France; Hospital Pitie-Salpetriere, Paris, France; Ospedale di Careggi, Firenze, Italy; Hopital La Miletrie, Poitiers, France; University Hospital, Uppsala, Sweden; Ankara University Faculty of Medicine, Ankara, Turkey; University Regensburg, Regensburg, Germany; University Hospital, Lund, Sweden; Centre Henri Becquerel, Rouen, France; Hospital U. Marqués de Valdecilla, Santander, Spain; VU University Medical Center, Amsterdam, Netherlands, The; Bristol Royal Hospital for Children, Bristol, United Kingdom; Royal Victoria Infirmary, Newcastle-Upon-Tyne, United Kingdom; University of Liege, Liege, Belgium; University of Saarland, Homburg, Germany; Istituto Scientifico H.S. Raffaele, Milano, Italy; Department of Haematology, Oxford, United Kingdom; Philipps Universitaet Marburg, Marburg, Germany; UnitT de Transplantation MTdullaire, Vandoeuvre Les Nancy, France; Rome Transplant Network, Rome, Italy; Rikshospitalet, Oslo, Norway; Royal Free Hospital and School of Medicine, London, United Kingdom; Glasgow Royal Infirmary, Glasgow, United Kingdom; Ospedale San Martino, Genova, Italy; Klinik für Knochenmarktransplantation, Idar-Oberstein, Germany; Hopitaux Universitaires de Geneve, Geneva, Switzerland; Johannes-Gutenberg-University, Mainz, Germany; Imperial College, London, United Kingdom; CHRU, Service des Maladies du Sang, Angers, France; Medizinische Universität Wien, Vienna, Austria; Haematology Department, Leeds, United Kingdom; Sahlgrenska University Hospital, Goeteborg, Sweden; Addenbrookes Hospital, Cambridge, United Kingdom; Southampton General Hospital, Southampton, United Kingdom; Bone Marrow Transplant Unit L 4043, Copenhagen, Denmark; Hospital Santa Creu i Sant Pau, Barcelona, Spain; University Hospital, Linköping, Sweden; Christie NHS Trust Hospital, Manchester, United Kingdom; Hospital Clínico, Salamanca, Spain; Dept. Haematology and Stem Cell Transplant, Budapest, Hungary; Institut Jules Bordet, Brussels, Belgium; Institute of Hematology and Blood Transfusion, Prague, Czech Republic; Centre Hospitalier Universitaire de Rennes, Rennes, France; Hadassah University Hospital, Jerusalem, Israel; Charité - Campus Benjamin Franklin, Berlin, Germany; Fédération de Greffe de Moelle et de, Clermont-Ferrand, France; Div. Stem Cell Transplantation and Immunotherapy, Kiel, Germany; Royal Liverpool University Hospital, Liverpool, United Kingdom; Ospedale San Gerardo, Monza, Italy; ICO – Hospital Duran i Reynals, Barcelona, Spain; North Trent BMT Programme (Adults), Sheffield, United Kingdom; Hôpital de l`ARCHET I, Nice, France; Vilnius University Hospital `Santariskiu Klinikos`, Vilnius, Lithuania; Manchester Royal Infirmary, Manchester, United Kingdom; S. Bortolo Hospital, Vicenza, Italy; University Hospital Maastricht, Maastricht, Netherlands, The; Leicester Royal Infirmary, Leicester, United Kingdom; Medical University of Gdansk, Gdansk, Poland; University Hospital, Zürich, Switzerland; Azienda Ospedaliera S. Giovanni, Torino, Italy; Umea University Hospital, Umeå, Sweden; Ospedale di Niguarda Ca` Granda, Milano, Italy; University Hospital, Udine, Italy; Medical University Graz, Graz, Austria; Hôpital Percy, Clamart, France; St. Bartholomew`s and The Royal London Hospital, London, United Kingdom; Turku University, Turku, Finland; Ospedale V. Cervello, Palermo, Italy; A.Z. Sint-Jan, Brugge, Belgium; Universita degli Studi di Bari, Bari, Italy; Ospedale La Maddalena - Dpt. Oncologico, Palermo, Italy; Ospedale Ferrarotto, Catania, Italy; University Medical Center Groningen (UMCG), Groningen, Netherlands, The; Tel Aviv Sourasky Medical Center, Tel Aviv, Israel; Ospedale Civile, Pescara, Italy; Ospedale Maggiore di Milano, Milano, Italy; Birmingham Heartlands Hospital, Birmingham, United Kingdom; RP Group Royal Perth Hospital, Perth, Australia; University of Napoli, Napoli, Italy; Universitätsklinikum Göttingen, Gottingen, Germany; Hospital Universitari Germans Trias i Pujol, Barcelona, Spain; University Hospital Erlangen, Erlangen, Germany; Hopital Nord, Saint Etienne Cedex 2, France; Elisabethinen-Hospital, Linz, Austria; University of Wales, Cardiff, United Kingdom; Institut Gustave Roussy, Villejuif, France; Hospital Clinic, Barcelona, Spain; University of Cologne, Cologne, Germany; Hacettepe University, Ankara, Turkey; University Hospital, Olomouc, Czech Republic; Hospital Universitario La Fe, Valencia, Spain; University Hospital Brno, Brno, Czech Republic; Evangelismos Hospital, Athens, Greece; Constantiaberg Medi-Clinic, Cape Town, South Africa; Central Clinical Hospital, Warsaw, Poland; AK St. Georg., Hamburg, Germany; Heinrich Heine Universität, Düsseldorf, Germany; King Hussein Cancer Centre, Amman, Jordan; Centre Hospitalier Universitaire, Caen, France; Hospital Vall d`Hebron, Barcelona, Spain; Ege University Medical School, Bornova-Izmir, Turkey; University Med. Center, Ljubljana, Slovenia; CHU Morvan, Brest, France; Institute of Haematology and Blood Transfusion, Warsaw, Poland; Charles University Hospital, Hradec Králové, Czech Republic; Academisch Ziekenhuis bij de Universiteit, Amsterdam, Netherlands, The; Belfast City Hospital, Belfast, United Kingdom; Inst. Portugues de Oncologia do Porto, Porto, Portugal; Universita Cattolica S. Cuore, Rome, Italy; K. Marcinkowski University of Medical Science, Poznan, Poland; Rambam Medical Center, Haifa, Israel; Policlinico G.B. Rossi, Verona, Italy; SPb State I. Pavlov Medical University, St. Petersburg, Russia; H SS. Antonio e Biagio, Alessandria, Italy; Klinikum Augsburg, Augsburg, Germany; DCTK, Wroclaw, Poland; Canterbury Health Laboratories, Christchurch, New Zealand; Hosp. Reina Sofia, Córdoba, Spain; Friedrich-Schiller-Universität Jena, Jena, Germany; Azienda Ospedaliera, Reggio Calabria, Italy; Hospital Gregorio Marañón, Madrid, Spain; Patras University Medical School, Patras, Greece; Univ. Est. de Campinas/TMO/UNICAMP, Campinas, Brazil; University Hospital VUB, Brussels, Belgium; Hospital Clínico Universitario, Valencia, Spain; Antwerp University Hospital (UZA), Antwerp Edegem, Belgium; Azienda Ospedali Riuniti di Ancona, Ancona-Torrette, Italy; University Hospital Gent, Gent, Belgium; Plymouth Hospitals NHS Trust, Plymouth, United Kingdom; Inst. Portugues Oncologia, Lisboa, Portugal; Hospital Covadonga, Oviedo, Spain; Robert-Bosch-Krankenhaus, Stuttgart, Germany; Ernst-Moritz-Arndt-Universität Greifswald, Greifswald, Germany; Klinikum Rechts der Isar, Munich, Germany; Hospital Morales Meseguer, Murcia, Spain; Hospital `Virgen del Rocio`, Sevilla, Spain; Western General Hospital, Edinburgh, United Kingdom; ZNA, Antwerp, Belgium; Beilinson Hospital, Petach-Tikva, Israel; Hospital Aranzazu, San Sebastian, Spain; Dipartimento di Oncologia, dei trapianti e delle, Pisa, Italy; Ospedale S. Camillo-Forlanini, Rome, Italy; Hospital San Maurizio, Bolzano, Italy; Allogeneic Stem Cell Transplant Center, Würzburg, Germany; Baskent University Hospital, Adana, Turkey; University Hospital, Bratislava, Slovakia; Tartu University Hospital, Tartu, Estonia; St. George`s Hospital, London, United Kingdom; Guy`s Hospital, London, United Kingdom; Karadeniz Technical University, Trabzon, Turkey; Policlinico Le Scotte, Siena, Italy; Hospital de Gran Canaria `Dr Negrin`, Las Palmas De Gran Canaria, Spain; AZ. Spedali Civili- Brescia Universitu of Brescia, Brescia, Italy; Hospital de la Princesa, Madrid, Spain; Specialized Children`s Oncohematpology Hospital, Sofia, Bulgaria; Hospital del SAS, Cádiz, Spain; Haukeland University Hospital, Bergen, Norway; University Hospital Innsbruck, Innsbruck, Austria; Hospedale Nord, Taranto, Italy; Ankara Numune Education and, Ankara, Turkey; Medical University of Lublin, Lublin, Poland; Ospedale A. Businco, Cagliari, Italy; IRCCS, Casa Sollievo della Sofferenza, San Giovanni Rotondo, Italy; Az. Ospedaliera S. Croce e Carle, Cuneo, Italy; Clinica Puerta de Hierro, Madrid, Spain; Kliniken Essen Süd, Essen, Germany; NHS Grampian, Aberdeen, United Kingdom; Fundeni Clinical Institute, Bucharest, Romania; Uni. Modena, Policlinico, Modena, Italy; KLINIKUM BREMEN - MITTE, Bremen, Germany; Mazzoni Hospital, Ascoli Piceno, Italy; Martin-Luther-Universität Halle-Wittenberg, Halle, Germany; Pesaro Hospital, Pesaro, Italy; Istanbul Tip Fakueltesi, Istanbul, Turkey; Wroclaw Medical University, Wroclaw, Poland; Medical Park Hospitals, Antalya, Turkey; Military Medical Academy, Belgrade, Serbia and Montenegro; University of Milano, Milano, Italy; Shariati Hospital, Teheran, Iran; A.O.R.N. `SAN.G MOSCATI`, Avellino, Italy; Unità Operativa Oncoematologia Pediatrica, Pisa, Italy; P.O. `R. Binaghi`, Cagliari, Italy; Military Medical Academy, Warsaw, Poland; University of Medicine and Pharmacy, Timisoara, Romania; GATA BMT Center, Ankara, Turkey; Universität Rostock, Rostock, Germany; Osmangazi University, Fac. of Medicine, Eskisehir, Turkey; Sectia Clinica de Hematolgoie si, Targu-Mures, Romania; The Trustee of London Clinic, London, United Kingdom; Istituto Clinico Humanitas, Milano, Italy; Ankara Oncology Research & Education Hospital, Ankara, Turkey; Hospital Universitario Son Espases, Palma De Mallorca, Spain; Cerrahpasa Medical School, Istanbul, Turkey; Hospital Univ. Virgen de las Nieves, Granada, Spain; National Haematology Centre, Riga, Latvia; Hospital Ampang, Ampang, Malaysia; Klinikum Oldenburg, Oldenburg, Germany; Campus Charité Mitte, Berlin, Germany; Univ. of Torino, Torino, Italy; American University of Beirut, Beirut, Lebanon; Ankara Bayindir Hospital, Haematology BMT, Ankara, Turkey; Hospital de Navarra, Pamplona, Spain; Wellington Hospital, Wellington, New Zealand; Belorussian Centre for Paediatric, Minsk, Belarus; Heilig Hartziekenhuis, Roeselare, Belgium; Hospital C. Panico, Tricase (Lecce), Italy; Arcispedale S. Maria Nuova, Reggio Emilia, Italy; Medical Academy, Wroclaw, Poland; CHRU Limoges, Limoges, France; Clinic Frankfurt (Oder) GmbH of Internal Medicine, Frankfurt (Oder), Germany; Hospital Universitario Virgen de la Arrixaca, Murcia, Spain; Ospedale Civile SS. Giovanni e Paolo, Venezia, Italy; Alfred Hospital, BMT Programme, Melbourne, Australia; Schneider Children`s Medical Center of Israel, Petach-Tikva, Israel; Institute G. Gaslini, Genova, Italy; Ospedale San Gerardo, Monza, Italy; Klinikum Karlsruhe gGmbH, Karlsruhe, Germany; IRCCS Policlinico San Matteo, Pavia, Italy; Hospital Clinico Universitario, Santiago De Compostela, Spain; Hospital de Santa Maria, Lisboa, Portugal; St.Savas Oncology Hospital, Athens, Greece; Univ. di Palermo, Palermo, Italy; Azienda Ospedaliera Universitaria San Martino, Genova, Italy; Klinikum der Johann-Wolfgang Goethe Universität, Frankfurt am Main, Germany; C.H.U. Timone Enfants, Marseille, France; Univ. of Parma, Parma, Italy; Clinica di Oncoematologia Pediatrica, Padova, Italy; University Hospital Center Rebro, Zagreb, Croatia; CHNDRF, Charleroi, Belgium; University Hospital Motol, Prague, Czech Republic; University of Cape Town Faculty of Health Sciences, Cape Town, South Africa; Istituto per l`Infanzia `Burlo Garofolo`, Trieste, Italy; University Hospital, Tübingen, Germany; Jagiellonian University, Krakow, Poland; University Children`s Hospital, Graz, Austria; Cardarelli Hospital, Napoli, Italy; Hospital Ramon y Cajal, Madrid, Spain; Clínica Universitaria de Navarra, Pamplona, Spain; IHOP, Lyon, France; Ospedale San Carlo, Potenza, Italy; Kreiskrankenhaus Hameln, Hameln, Germany; Hospital Guglielmo da Saliceto, Piacenza, Italy; Albert Aberts Stem Cell Transplant Unit, Pretoria, South Africa; Adnan Menderes University Med. Faculty, Aydin, Turkey; Spedali Civili - Brescia, Brescia, Italy; European Institute of Oncology, Milano, Italy; Hospital Juan Canalejo, La Coruña, Spain; ITMO-Instituto de Transplante de Medula Osea, La Plata, Argentina; St. Anna Kinderspital, Vienna, Austria; Chaim Sheba Medical Center, Tel-Hashomer, Israel; Hospital Carlos Haya, Málaga, Spain; Pediatric University Teaching Hospital, Bratislava, Slovakia; Dokuz Eylül Universitesi, Izmir, Turkey; Royal Hospital for Sick Children, Glasgow, United Kingdom; Musgrove Park Hospital (Somerset), Taunton, United Kingdom; The Children`s Hospital at Westmead, Sydney, Australia; Marmara University, Istanbul, Turkey; Niño Jesus Children`s Hospital, Madrid, Spain; University of Jena, Jena, Germany; University Hospital, Collegium Medicum UMK, Bydgoszcz, Poland; Royal Liverpool Children`s NHS Trust, Liverpool, United Kingdom; Our Lady`s Hospital for Sick Children, Dublin, Ireland; University of Bologna, Bologna, Italy; Wilhelminenspital, Vienna, Austria; Pédiatrie et Génétique Médicale, Rouen, France.
